# Inequities in Antenatal Care, and Individual and Environmental Determinants of Utilization at National and Sub-national Level in Pakistan: A Multilevel Analysis

**DOI:** 10.15171/ijhpm.2017.148

**Published:** 2018-01-30

**Authors:** Ambreen Sahito, Zafar Fatmi

**Affiliations:** ^1^Department of Community Medicine, Isra University, Hyderabad, Pakistan.; ^2^Department of Community Health Sciences, Aga Khan University, Karachi, Pakistan.

**Keywords:** Inequity, Antenatal Care Utilization, Determinants, Multilevel Analysis, Pakistan

## Abstract

**Background:** Nationally representative surveys are vital for gauging progress in health and planning health services. However, often marred with inadequate analysis to provide any guidance to health policy and planning. Most recent Pakistan Demographic and Health Survey (PDHS) 2012-2013 is an inclusive nationally representative investigation. Nonetheless, its published report offers limited evidence regarding antenatal care (ANC). Furthermore, after 18th constitutional amendment, policies are principally made at provincial level in Pakistan; therefore, it is imperative to have contextual evidence at sub-national level to feed programs and policies.

**Methods:** We analysed 7142 women with a recent birth, to assess the individual and environmental determinants of ANC, adapting Andersen’s model of healthcare utilization, by multilevel analysis. Separate models of determinants were developed for the national level and five provinces using survey command in Stata version 12.1.

**Results:** Besides that the recommended ANC coverage (≥4 visits) is low in Pakistan (36%), gross inequities exist predominantly across provinces (12% to 82%). Small differences exist between urban and rural localities. Education, health literacy and socio-economic status of women were strong predictors, while communities with high concentration of literate women very strongly predict ANC use (odds ratio [OR] = 12). Determinants of ANC vary at national and at sub-national level. For example, women’s education had no influence on ANC utilization in Khyber Pakhtunkhwa (KPK) and Baluchistan (BC) provinces. Notably, husband’s education was significantly associated with ANC utilization in KPK only. Significant positive interaction exists between urban areas and larger provinces (Punjab, Sindh, and KPK). Also, very strong positive interaction occurs when women have secondary or particularly higher level of education and living in urban areas or larger provinces.

**Conclusion:** This study highlights conspicuous contextual differences which determine maternal care at national and sub-national level. It identified contextual factors which are important for planning maternal health services between and within provinces. High positive interaction for ANC utilization between women education, urban areas and larger provinces highlights the inequities which need to be addressed. It also identified factors at the community level (cluster) which relates to overall context and influence individual behavior and highlights the diminishing urban-rural gap in service utilization in Pakistan.

## Background

**Figure 1:**
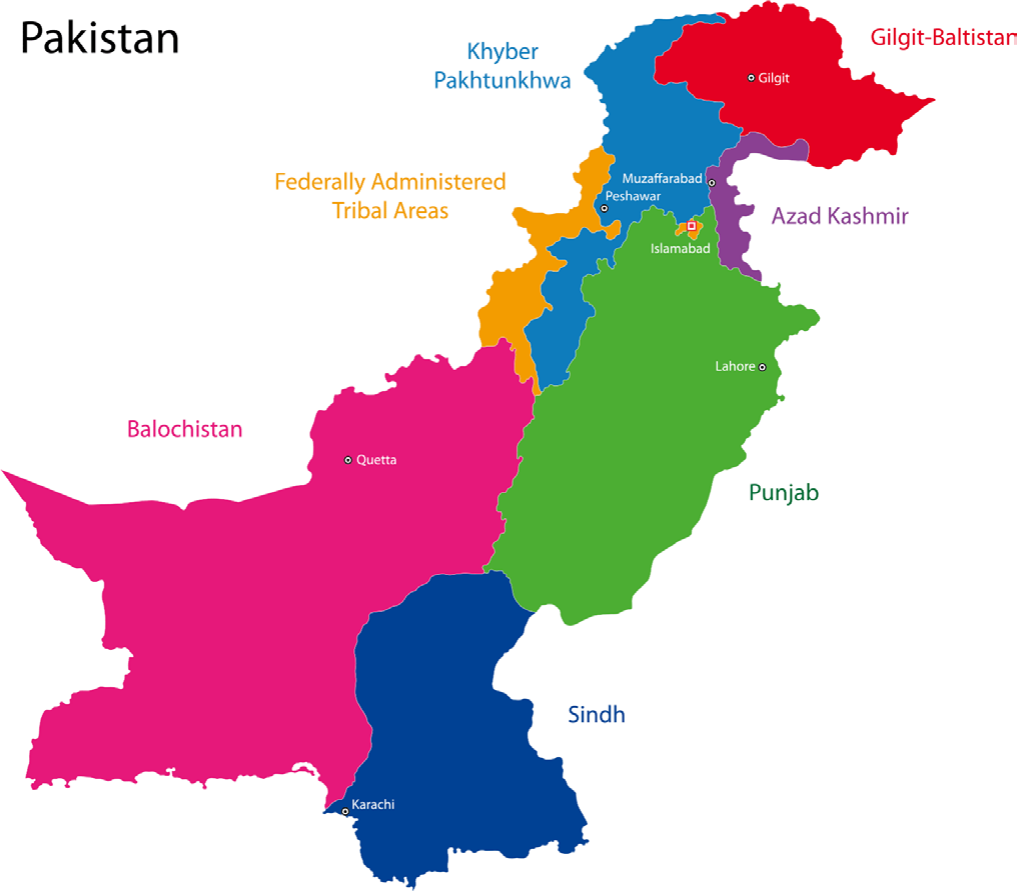
Map of Pakistan, Provinces and Federally Administered Territories.

Pakistan, is a large country comprising five provinces (Punjab, Sindh, Khyber Pakhtunkhwa [KPK], Baluchistan [BC], and Gilgit-Baltistan [GB]) and federally administered areas is home to 208 million populations (Figure 1).^1^ Recent estimates of maternal mortality ratio (MMR) was 276/100 000 live births, which was higher than neighboring regional countries except Afghanistan.^2^ Maternal health service provision and utilization was poor. Current estimates of antenatal care (ANC), skilled birth attendance (SBA) and post-natal care (PNC) utilization was 73% (even for one visit during pregnancy), 52% and 60%, respectively.^2^

ANC is a key indicator for maternal health services in a country. An estimated 74% of overall maternal deaths can be averted if every women had access to maternal healthcare services.^3,4^ ANC not only helps to identify and manage pregnancy risks but increases SBA, PNC, and was associated with better newborn care and greater knowledge of contraception.^5^ Despite its importance, in developing countries only 52% pregnant mothers receive recommended ANC (ie, four or more visits with the skilled provider during pregnancy).^6^ Although information usually exists in national surveys regarding what determines maternal health services, it has not been put into use due to inadequate analysis.

According to Pakistan Demographic and Health Survey (PDHS) 2012-2013, only 37% married women of reproductive age (MWRA) received recommended ANC (≥4 visits with the skilled providers).^7^ PDHS is an inclusive survey, however, has only provided descriptive statistics about ANC coverage according to service provider, number of visits and differentials by women background characteristics and composition. Although the report highlights inequities in ANC but the information remain obscure and deceptive. It has potential for erroneous interpretation for programmatic planning and policy formulation. Furthermore, it has not explored the factors which influence the ANC utilization, thus not helpful for any suggestion for improvement in services.^7^ The report also suggests gross inequities in ANC utilization across regions and place of residence. For example, 87% of women living in urban areas received ANC as compared to 67% in rural areas. Similarly, in Islamabad Capital Territory (ICT) 97% of MWRA received ANC which was only 31% in BC.^7^ These results do not provide insight about why these differentials exist between these strata and regions and what needs to be done to improve these services in local provincial context. Furthermore, in 2010, an important constitutional reform was made in Pakistan, 18th amendment. This reform enables provinces to make their own policy and strategies.^8^ Disappointingly, evidence about determinants of ANC utilization does not exist that work in different context like rural/urban or determinants across regions and provinces for formulating evidence based policies in Pakistan.

ANC utilization is affected by many individual and context specific environmental factors.^9-12^ Nonetheless, few studies have considered beyond individual and household factors of healthcare utilization, worldwide. To efficiently feed policies, in addition to individual and household level factors, cultural differences, governance and local environmental determinants need to be studied which influence the success of healthcare programs. These factors, although always qualitatively felt to be important while planning the programme, are usually measured at community level and limited analysis of important national surveys do not provide insight to these aspects. Previous studies in Pakistan on determinants of ANC utilization were done only on small geographic areas.^13-16^ Thus were not helpful for informing policy-makers for programmatic success.

The current study utilizes the nationally representative sample from five provinces and capital region of Pakistan. Understanding factors that affect utilization of ANC services will help to develop policies and design strategies to improve service utilization in Pakistan in post 18th amendment context.

Keeping in view the gaps in information, we analyzed the most recent PDHS 2012-2013, to determine the factors that affect ANC utilization in Pakistan. We utilized adapted Anderson’s model for healthcare utilization (a well-studied and tested model)^17^ to analyze comprehensive set of variables which may be helpful for overall maternal healthcare programmatic implementation. Furthermore, we studied the factors of ANC utilization across provinces and rural and urban regions of Pakistan. Several factors, for example provincial differences in urban areas and education level of women and husband which operates synergistically (interaction) to influence ANC utilization, were studied together to inform researchers and policy-makers. In addition, we studied factors beyond household level which operates in the context of communities and overall environment using multilevel analysis.

## Methods

PDHS 2012-2013 was the third round of survey conducted under global Demographic and Health Survey (DHS) program under the National Institute of Population Studies (NIPS) and funded by the United States Agency for International Development (USAID).^7^

### Study Population and Sampling

Two stage stratified cluster sampling strategy was used to select a nationally representative (excluding Azad Jammu and Kashmir, federally administered tribal areas [FATA], and restricted military and protected areas) sample of 14 000 households. The total population of Pakistan has been divided into primary sampling units (PSUs), which are clusters of 250-300 households. Using population proportional to size (PPS) sampling scheme, 500 PSUs were selected in the first stage. In the second stage, 28 households were randomly selected from each PSU. Smaller geographic regions like urban areas of BC, ICT and GB were oversampled.

Among 12 943 households who gave consent for survey, a total of 13 558 MWRA were interviewed. We restricted our analysis to 7142 MWRA who gave birth to at least one child in the five years preceding the survey.

PDHS used four types of questionnaires: Household, woman, man, and community level questionnaire.

### Andersen’s Model of Healthcare Utilization

**Figure 2:**
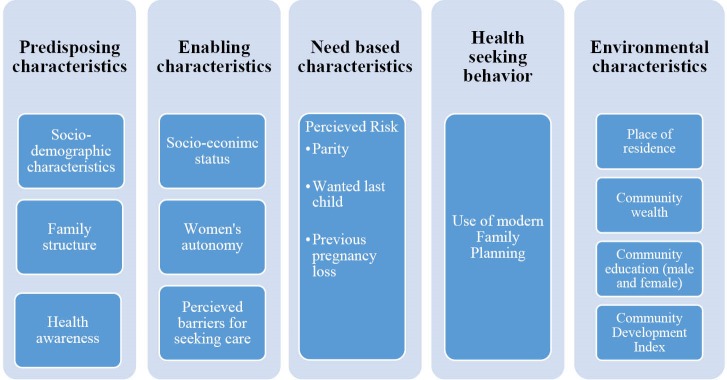
Adapted Andersen’s Model for Antenatal Care Utilization.

Andersen’s model was adapted to identify determinants of ANC.^17,18^ The Andersen’s model uses a systems perspective to integrate individual and environmental factors and classify them into: predisposing (existing characteristic of the individual/family which increases likelihood of use of health services), enabling (for example socioeconomic status, access to healthcare etc), need based characteristics (those who have existing morbidity), health seeking behaviour (those who generally are utilizers of services) and environmental factors (include community level factors which are context specific). Variables analyzed in our study were only taken from Woman’s questionnaire. (Variable description, classification and computation, is given in Table 1 and Figure 2).

**Table 1:** Weighted Univariate and Multivariate Analysis Showing Association of Factors With Recommended ANC Utilization Among Women (15-49 years) in Pakistan - PDHS 2012-2013 (n = 7142)

**Variable**	**Univariate**		**Multivariate**	
	**OR**	**(95% CI)**	**OR**	**(95% CI)**
**1. Predisposing Characteristics**				
Mean age (centred)	0.9	(0.9-1.0)	1.0	(0.9-1.0)
Mean age (squared)	0.9	(0.9-1.0)		
Age categories (y)				
15-19	1			
20-24	1.0	(0.6-1.5)		
25-29	1.1	(0.7-1.8)		
30-34	1.1	(0.7-1.9)		
35-39	0.6	(0.4-1.1)		
40-44	0.7	(0.3-1.4)		
45-49	0.4	(0.2-0.7)		
Women’s education				
No education	1		1	
Primary (1-5 years)	2.6	(2.1-3.3)	1.4	(1.0-1.8)
Secondary (6-10 years)	6.2	(5.0-7.7)	1.8	(1.4-2.4)
Higher (>10 years)	19.0	(13.2-27.3)	3.5	(2.3-5.2)
Husband’s education				
No education	1			
Primary (1-5 years)	1.5	(1.2-2.0)		
Secondary (6-10 years)	3.0	(2.4-3.7)		
Higher (>10 years)	5.8	(4.6-7.3)		
Household size				
More than 10	1		1	
7-10 members	1.1	(0.9-1.3)	1.0	(0.8-1.3)
6 or less	1.5	(1.3-1.9)	1.3	(1.0-1.7)
Number of under-5 children (mean, SD)	0.9	(0.8-0.9)	0.9	(0.8-1.0)
Health literacy				
Low	1		1	
High	4.2	(3.6-4.8)	1.4	(1.2-1.7)
Exposure to media (TV/radio/newspaper) (mean, SD) (per unit increase)	1.9	(1.8-2.0)	1.1	(1.0-1.2)
Heard of family planning on media		
No	1		1	
Yes	2.5	(2.1-3.1)	1.2	(1.0-1.5)
**2. Enabling Characteristics**				
Wealth index				
Poorest	1		1	
Poorer	1.7	(1.4-2.3)	1.5	(1.1-2.0)
Middle	2.9	(2.2-3.8)	1.9	(1.4-2.6)
Richer	6.5	(5.0-8.4)	2.8	(2.0-4.0)
Richest	22.7	(17.3-29.8)	5.4	(3.7-7.9)
Own transport				
No	1		1	
Yes	2.5	(2.2-2.9)	1.2	(1.0-1.4)
Husband’s occupation				
Not employed/unskilled	1			
Skilled/non-manual	2.4	(2.0-2.9)		
Professional/technical	3.6	(2.7-4.7)		
Women’s employment status				
Not employed	1			
Employed	1.0	(0.9-1.3)		
Residing with husband				
No	1			
Yes	1.0	(0.8-1.3)		
Women’s autonomy				
Low	1			
High	1.1	(0.9-1.2)		
Getting permission				
Problem	1			
Not a problem	1.0	(0.8-1.2)		
Getting money for treatment				
Problem	1			
Not a problem	1.0	(0.8-1.2)		
Distance to health facility				
Problem	1			
Not a problem	1.0	(0.8-1.2)		
Having to take transport				
Problem	1			
Not a problem	0.9	(0.8-1.0)		
Going alone for treatment				
Problem	1			
Not a problem	0.9	(0.8-1.0)		
**3. Need Based Characteristics**				
Parity				
5 or more children	1		1	
3-4	1.8	(1.5-2.1)	1.0	(0.8-1.3)
2 or less	2.7	(2.3-3.2)	1.5	(1.1-1.9)
Wanted child				
No	1			
Yes	1.0	(0.9-1.2)		
Pregnancy loss				
No	1		1	
Yes	1.2	(1.0-1.3)	1.5	(1.3-1.8)
**4. Health Seeking Behaviour**				
Use of family planning method				
No/traditional method	1			
Modern	1.0	(0.9-1.2)		
**5. Environmental Factors**				
Region				
BC	1		1	
KPK	2.2	(1.4-3.5)	1.7	(1.2-2.6)
Gilgit Baltistan	3.1	(1.6-5.9)	4.4	(2.4-8.1)
Punjab	4.3	(2.8-6.6)	2.1	(1.4-3.0)
Sindh	5.6	(3.6-8.8)	3.7	(2.5-5.4)
ICT	32.5	(19.8-53.4)	6.1	(3.9-9.4)
Place of residence				
Rural	1		1	
Urban	4.8	(3.8-6.0)	1.4	(1.2-1.8)
Community development index				
Low	1			
High	5.4	(4.4-6.6)		

Abbreviations: ICT, Islamabad Capital Territory; BC, Baluchistan; SD, standard deviation; OR, odds ratio; ANC, antenatal care; KPK, Khyber Pakhtunkhwa.

### Data Analysis and Procedures

Data was analyzed in Stata version 12.1 by using survey command for complex survey design. Descriptive statistics including weighted proportions with 95% CI for categorical variables and mean along with standard deviation (SD) for continuous variables was calculated for the overall sample and for those utilizing recommended ANC (4 or more visits) versus less than recommended use (3 or less visits). Sampling weights were calculated based on sampling probabilities for each sampling unit ie, PSU (see details in PDHS 2012-2013).^7^ With the available data we estimated ANC utilization at national level and separately for five provinces (BC, GB, KPK, Punjab, and Sindh) and urban and rural areas.

**Box 1.** Definition of Variables, Development and its Computation Used in the Analysis

**Outcome variable:** ANC: Women who during the recent pregnancy visited any formal healthcare provider for a minimum of four times were considered to have recommended ANC.

**Independent variables:**

Level 1 variables: Individual/household level variables and their formulation

Explanatory variables for ANC were grouped according the Andersen’s model, as follows:

*1. Predisposing characteristics:*

a. Socio-demographic characteristics: Women’s age was categorized into reproductive age groups for descriptive statistics. However, mean age (centred) was used for adjustment in multivariable analysis. Women’s and husband’s education were categorized into none, primary, secondary, and higher education.
b. Family structure: Total number of household members (household size) were categorized as ‘more than 10 vs. 7-10 vs. 6 or less’ and total (mean) number of under-5 children in a household.
c. Health awareness:
I - Health literacy (*): Women’s knowledge about common diseases (hepatitis, tuberculosis, HIV/AIDS and sexually transmitted infections). Each answer were scored as ‘yes’ (=1) and ‘no’ (=0) and principal component analysis (PCA) was conducted to formulate composite variable of health literacy. PCA output was normalized and converted into binary variable of ‘low vs. high’ using median cut-off (50th percentile) for analysis.
II- Exposure to media: Frequency of listening to radio, watching television and reading newspaper were recorded on ordinal scale of 0-2: 0 being never, 1 as occasional or less than once per week and 2 as frequent or daily. We added scores of these variables to make a composite variable of media exposure, scaled 0-6. This was used as a continuous variable in the analysis.
III- Heard of family planning (on radio/TV/newspaper/magazine): On any media during last few months was recorded as ‘yes/no.’

*2. Enabling characteristics:*

a. Socioeconomic status: We used five variables as measure of socio-economic status ie, wealth index, woman’s employment status, husband’s employment, owning a motorized vehicle and residing with husband. Wealth index is comprehensive indicator developed in three steps from household ownership of assets using PCA. Wealth index is reported in PDHS report as wealth quintiles. Woman’s employment was explained as binary variable as ‘employed vs. Unemployed.’ Husband’s occupation was categorized as ‘unemployed/unskilled vs. skilled/non-manual vs. professionals/technical/manger.’ Ownership of motorized vehicle (motorcycle/car/truck) in the house as transport was also categorized as a binary (yes/no) variable. ‘Residing with husband or alone’ was also considered as a variable of socio-economic status.
b. Women’s autonomy (*): Women’s involvement in decision making in the household was used as a proxy for autonomy. Three questions regarding decision-making were used: women’s health seeking, large household purchases and visits to family or relatives. All these responses were categorized as ‘partner/someone else decides vs. respondent with partner/ other member vs. respondent alone.’ We ran PCA to come up with a composite variable which was normalized to scale of 0-1 and converted into binary variable of ‘low vs. high’ using a cut-off of median (50th percentile).
c. We did PCA to generate an index based on three variables: community access to improved water, availability of electricity and type of fuel (biomass vs non-biomass) used for cooking in the household.
d. Perceived barriers for seeking care (*): We used set of questions to assess perceived barriers for seeking care. These questions included ‘going alone to a health facility,’ ‘getting permission for going to health facility,’ ‘getting money required for healthcare,’ and ‘distance to the health facility.’ These variables were assessed separately for its influence on recommended ANC use. At individual level we analysed all these questions separately but to see barriers at environmental (contextual) level we developed a composite variable by running PCA of all these questions (as above).

*3. Need based characteristics (perceived need due to biological risk):*

For perceived risk three variables were used including parity (5 or more vs. 3-4 vs. 2 or less), wanted last child (yes/no) and any previous pregnancy loss (yes/no).

*4. Health seeking behaviour:*

Use of modern family planning method was used as proxy indicator for women’s health seeking behaviour (no method/traditional method vs. modern family planning method).

**Level 2 (cluster) variables: **

*5. Environmental (contextual) factors*

Environmental level variables were created as averages or proportions by aggregating individual information in cluster. Clusters were 500 primary sampling units (PSUs), comprising 248 enumeration blocks in urban and 252 mouzas/dehs/villages in rural areas.

Place of residence (urban vs. rural), community wealth (mean of wealth index), community woman’s education (proportion of woman in ‘none vs. primary vs. secondary vs. higher education’), community husband’s education (same as woman’s education), community development index (CDI) were considered as environmental (contextual) factors to influence recommended ANC utilization.

Community Development Index (*): We did PCA to generate an index based on three variables: community access to improved water, availability of electricity and type of fuel (biomass vs nonbiomass) used for cooking in the household.

* Health literacy, women’s autonomy, perceived barriers for seeking care and CDI were composite indicators derived by PCA method. For PCA, first component was retained in all cases. First component of health literacy explained 54.6% of variance with eigenvalue of 2.18. With respect to composite indicator CDI’s first component explained 45.5% of variance with eigenvalue of 1.36, while for autonomy 77.2% of variance was explained by component 1 with eigenvalue of 2.3. We rescaled values for all these composite indicators on continuous scale from 0-1 and converted to binary variables ‘low and high’ using median (50th percentile). All of these indicators were compared with stillbirth rate and postnatal care to test their validity.

Weighted univariate logistic regression was done to calculate odds ratio (OR) and 95% CI to initially explore the factors which might influence recommended ANC utilization using the framework of Anderson’s model (described above).

For multivariate analysis, we developed separate models to determine independent factors for recommended ANC utilization each for: (1) overall sample population (national); (2) urban population; (3) rural population; and (4) 5 provinces. Variables with *P* value <0.25 at univariate level were considered for multivariable models to estimate adjusted OR (AOR). Variables were checked for multicollinearity. Interaction was checked between provinces, urban/rural areas and educational level of woman and husband. Backward elimination procedure was used to remove least significant variable in the model and this procedure was continued to develop parsimonious model.

For the multilevel analysis, we developed a model for the overall (national) sample to see the influence of environmental (cluster) factors on individual health seeking behavior for recommended ANC.^19^ We assessed variables that were significantly associated with recommended ANC utilization in the multivariable model (level 1 variables – individual/household level). Place of residence (urban/rural, province) were used for contextual variables. In addition, at the cluster (PSU) level we determined the proportion of various level of education for women and husband at the cluster level, mean wealth was derived from averages of the individual wealth index and proportion of CDI as ‘low and high’ was considered as contextual/environmental determinants (level 2 variables – community level). Three sequential models including random intercept were tested: (*i*) Model 0 (null or empty model) – did not include explanatory variables and observed the effects of the environmental level variables on the propensity to use recommended ANC; (*ii*) Model 1 – controlled for maternal characteristics and variables related to her partner and house­hold (level 1 variables- individual/household level characteristics) were included to determine the extent to which cluster level differences were explained by individual/household characteristics of the clusters; and (*iii*) Model 2, in which the environmental level (level 2 –cluster level variables) variables were added to investigate whether this contextual phenomenon was influenced by specific cluster characteristics. We used Goldstein and Snijders approaches (the latent variable method) to estimate the effect at individual level and cluster (area) level components.^20,21^

These multilevel models allowed us to explore variations attributed to individual/household char­acteristics and the amount attributed to the cluster level variables. The relationship between variances was expressed using the variation partition coefficient (VPC), which measures the proportion of total variance that is attributed to between-group dif­ferences. The VPC ranges from 0 to 1, where 0 indicates that there are no between-group differ­ences and 1 indicates there are no within-group differences. Multilevel analysis was done without applying weights and survey commands, since weighted multilevel analysis procedures have not been rationalized yet.^22,23^

## Results

**Figure 3:**
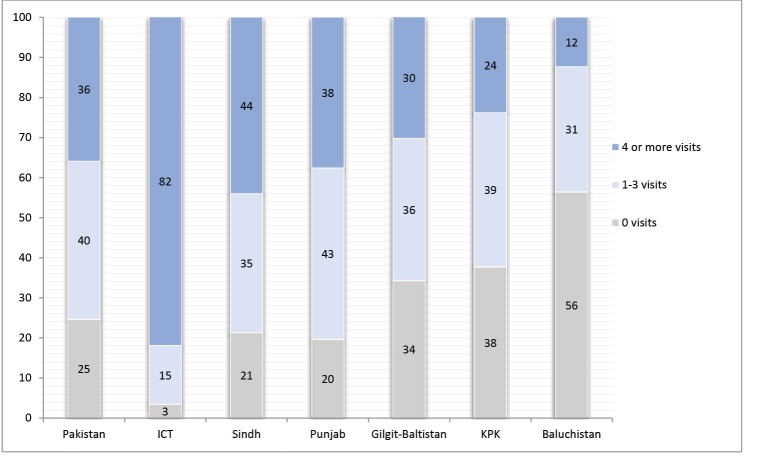
Proportion of Women Received Antenatal Care (ANC) at National, Provincial, and Regional Level (PDHS-2012-2013).

Gross inequities exist in ANC utilization across provinces and urban/rural areas. As shown in Figure 3, overall 36% MWRA received recommended ANC (4 or more visit during pregnancy) in Pakistan. Region-wise recommended ANC utilization was highest in ICT (82%), while only 12% MWRA received recommended care about 56% receive no ANC in BC province.

Descriptive characteristics of the sample according to recommended use of ANC (≥4 visits) and less than recommended ANC use (≤3 visits) is provided in Supplementary file 1 (Table S1). Table 1 shows weighted univariate and AOR along with 95% CI for factors associated with recommended ANC utilization at the national level (overall sample). The variables were categorized according to the Anderson’s model (described above).

Table 1 shows ANC determinants, at national level and Table S2 shows results according to rural and urban areas, at multivariable analysis. Women education, smaller family (number of under-5 children)/household size, higher health literacy and exposure to media increased the recommended ANC utilization among women in Pakistan (overall sample). Educated women were more likely to receive recommended ANC and higher odds was observed from primary, secondary to higher education. Women education increased recommended ANC in both urban and rural areas, however this effect was stronger in urban areas. Smaller household size (≤6 vs. >10 members) increased recommended ANC utilization 1.5 times in rural areas, while with each decreasing number of under five children (smaller family) there was an increase in recommended ANC utilization (see Supplementary file 1, Table S2). MWRA belonging to high socioeconomic status more likely to receive ANC, richest wealth quintile were six times more likely to receive recommended ANC compared to poorest quintile. Those owned transport were more likely to receive recommended ANC. Among need-based characteristics, those with low parity and had previous pregnancy loss were more likely to receive recommended ANC. Women residing in GB, Sindh, Punjab, and KPK were more likely to seek ANC as compared to women living in BC, and these differences were more pronounced in urban as compared to rural areas (see Supplementary file 1, Table S2).

Along with multivariate model in overall sample (Pakistan), we found strong interaction between education of woman and husband, urban localities of large provinces (see Supplementary file 1, Table S3). Those women living urban areas of Punjab, Sindh and KPK had synergistic effect of recommended ANC utilization compared to those living in rural areas and BC province. Women who were educated to secondary and particularly higher level and living in provinces of Punjab, Sindh, KPK or region of ICT and GB had synergistic positive association for recommended ANC utilization compared to those not educated and living in BC. Similar interaction existed between higher woman education and urban localities for increase recommended ANC use compared to no education and rural localities. While, not for urban areas, but only husband’s higher education and living in large provinces had synergistic positive association for recommended ANC utilization. Interestingly, women with high education level and husband with primary education had strong negative interaction (OR; 0.1) compared to if both husband and wife had no education. Table 2 shows ANC determinants at multivariable analysis in different regions of Pakistan. Higher women education increased ANC utilization in all regions except BC and KPK, and husband’s higher education increased ANC utilization in KPK only. However, smaller household size increased ANC utilization in Sindh only but with increasing number of each under-5 children in household decreased ANC utilization in BC, GB and Punjab. Increased exposure to media increased ANC utilization in KPK (OR; 1.2) only. Higher health literacy predisposed women for receiving ANC in all regions (OR; 1.5-2.4) except KPK while family planning awareness increased ANC in BC and KPK (OR; 4.6 and 2.4, respectively) only. In Punjab MWRA living in urban areas were 1.5 times likely to receive ANC. Higher SES enabled ANC utilization in all regions. Richest MWRA were 4-7 times more likely to receive ANC. Residing with husband decreased odds of receiving ANC in KPK only (OR;  0.7). Husband occupation as skilled worker/professional and owning a transport enabled females for receiving ANC in Sindh only (OR: 1.4 and 1.6, respectively). Women who had 2 or less children were more likely to receive ANC in all regions except GB. Women with history of pregnancy loss were likely to receive ANC in KPK, Sindh, and Punjab (OR; 1.4, 1.7, and 1.5, respectively). Regarding perceived barriers for not seeking care, distance to health facility was significantly associated with receiving ANC in BC. MWRA who reported distance to health facility was not a problem were 2.1 times more likely to receive ANC.

**Table 2:** Weighted Multivariate Analysis Depicting Factors for Recommended ANC Utilization Among Women in Pakistan Stratified According to Provinces – PDHS 2012-2013

Multivariate Variable	**BC (n = 1107)**		**GB (n = 699)**		**KPK (n = 1477)**		**Sindh (n = 1531)**		**Punjab (n = 1844)**	
	**OR**	**95% CI**	**OR**	**95% CI**	**OR**	**95% CI**	**OR**	**95% CI**	**OR**	**95% CI**
**1. Predisposing Characteristics**										
Mean age (squared)	**-**	**-**	**-**	**-**	**-**	**-**	**-**	**-**	1.0	(0.9-1.1)
Women’s education		-			-	-				
No education	-		1				1		1	
Primary (1-5 years)			3.2	(1.0-10.4)			1.4	(0.9-2.2)	1.4	(1.0-2.1)
Secondary (6-10 years)			5.3	(2.6-10.8)			2.1	(1.3-3.2)	2.1	(1.5-2.9)
Higher (>10 years)			2.5	(1.2-5.4)			6.6	(3.2-13.6)	4.2	(2.3-7.9)
Husband education		-		-			-	-	-	-
No education	-				1					
Primary (1-5 years)			-		1.0	(0.6-1.9)				
Secondary (6-10 years)					2.0	(1.4-2.8)				
Higher (>10 years)					1.9	(1.2-3.1)				
Household size		-		-	-	-			-	-
More than 10	-						1			
7-10 members			-				1.7	(1.1-2.5)		
6 or less							2.1	(1.4-3.1)		
Under-5 children (per child increase)	0.8	(0.7-0.9)	0.8	(0.6-0.9)	-	-	-	-	0.9	(0.8-1.0)
Health literacy						-				
Low (first tertile)	1		1		-		1		1	
High	2.3	(0.9-5.5)	2.4	(1.2-4.6)			1.7	(1.2-2.4)	1.5	(1.1-1.9)
Exposure to media (TV/radio/newspaper) (per unit increase)	0.8	(0.6-0.7)	-	-	1.2	(1.0-1.5)	-	-	-	-
Heard of family planning on media			-	-			-	-	-	-
No	1									
Yes	4.6	(2.3-9.3)			2.1	(1.4-3.1)				
**Enabling Characteristics**										
Wealth quintile										
Poorest	1		1		1		1		1	
Poorer	1.5	(0.7-3.3)	2.1	(1.1-3.8)	1.7	(0.9-3.2)	1.5	(1.0-2.4)	1.6	(0.9-2.8)
Middle	2.1	(0.7-6.3)	1.4	(0.7-3.0)	2.0	(1.0-4.0)	2.3	(1.5-3.5)	2.1	(1.2-3.7)
Richer	1.5	(0.6-3.6)	4.8	(1.9-11.9)	3.3	(1.5-7.1)	3.3	(2.1-5.3)	3.2	(1.8-5.7)
Richest	3.8	(0.9-15.0)	3.5	(0.3-36.1)	7.7	(3.6-16.5)	5.8	(3.2-10.7)	6.0	(3.3-11.0)
Own transport (Motorcycle/car)			-	-	-	-			-	-
No	-	-					1			
Yes							1.6	(1.2-2.2)		
Husband occupation		-	-	-	-	-			-	-
Not employed/unskilled	-						1			
Skilled/non-manual							1.4	(1.0-1.9)		
Professional/technical							1.2	(0.7-2.0)		
Residing with husband		-	-	-			-	-	-	-
No	-				1					
Yes					0.7	(0.5-1.0)				
Distance to health facility			-	-	-	-	-	-	-	-
Problem	1									
Not a problem	1.1	(0.6-2.0)								
**Need Based Characteristics**										
Parity			-	-						
5 or more children	1				1		1		1	
3-4	1.4	(0.7-2.8)			1.4	(1.0-2.0)	0.8	(0.5-1.1)	1.2	(0.9-1.7)
2 or less	2.1	(1.3-3.6)			2.2	(1.6-3.0)	1.6	(1.1-2.2)	1.5	(1.0-2.1)
Pregnancy loss			-	-						
No	-	-			1		1		1	
Yes					1.4	(1.1-2.0)	1.7	(1.3-2.2)	1.5	(1.2-1.9)
Environmental factors										
Place of residence	-		-	-	-	-	-	-		
Rural		-							1	
Urban									1.5	(1.2-2.0)

Abbreviations: ICT, Islamabad Capital Territory; SD, standard deviation; OR, odds ratio; ANC, antenatal care; PDHS, Pakistan Demographic and Health Survey; BC, Baluchistan; KPK, Khyber Pakhtunkhwa; GB, Gilgit-Baltistan. *ICT was not analysed separately due to small sample size.

Table 3 shows results of multilevel logistic regression model. Null model shows that 32.4% of variance in the probability of seeking recommended four ANC visits is due to differences in clusters and thus may be explained by environmental level variables. Variance decreased to 20% after including individual level variables (Model 1) and it decreased to 14.7% after including environmental level variables (Model 2).

**Table 3:** Multilevel Analysis Showing Models With (Model 2) and Without (Model 1) Environmental Level Factors Associated With ANC Utilization in Pakistan (n = 7142)

**Variables**	**Model 1**		**Model 2**	
	**OR**	**95% CI**	**OR**	**95% CI**
**Individual Level Factors**				
Mean age (squared)	1.0	(1.0-1.0)	1.0	(1.0-1.0)
Women’s education				
No education	1		1	
Primary (1-5 years)	1.5	(1.2-1.8)	1.3	(1.1-1.6)
Secondary (6-10 years)	2.0	(1.6-2.4)	1.6	(1.3-2.0)
Higher (>10 years)	3.0	(2.3-4.0)	2.2	(1.7-2.9)
Household size				
More than 10	1		1	
7-10 members	1.2	(1.0-1.5)	1.2	(1.0-1.4)
6 or less	1.4	(1.1-1.7)	1.2	(1.0-1.5)
Under-5 children (per child increase)	0.9	(0.8-0.9)	0.9	(0.9-1.0)
Exposure to media (TV/radio/newspaper) (per unit increase)	1.1	(1.0-1.2)	1.1	(1.0-1.1)
Health literacy				
Low	1		1	
High	1.3	(1.1-1.7)	1.5	(1.2-1.9)
Heard of family planning on media				
No	1		1	
Yes	1.3	(1.1-1.5)	1.3	(1.1-1.5)
Wealth index				
Poorest	1		1	
Poorer	1.5	(1.2-1.9)	1.4	(1.1-1.8)
Middle	2.0	(1.6-2.6)	1.9	(1.4-2.4)
Richer	3.3	(2.5-4.2)	2.6	(2.0-3.4)
Richest	7.8	(5.8-10.4)	5.3	(3.8-7.3)
Parity				
5 or more children	1		1	
3-4	1.1	(0.9-1.3)	1.1	(0.9-1.3)
2 or less	1.7	(1.4-2.0)	1.7	(1.5-2.0)
Any pregnancy loss				
No	1		1	
Yes	1.4	(1.9-1.5)	1.3	(1.2-1.5)
Environmental Level Factors				
Region			1	
BC			1.4	(1.0-2.0)
KPK			3.3	(2.2-4.8)
GB			1.3	(0.9-1.9)
Punjab			3.1	(2.2-4.5)
Sindh			3.3	(2.1-5.3)
Place of residence				
Rural			1	
Urban			1.0	(0.8-1.2)
Community development index				
High (third tertile)			1	
Intermediate (second tertile)			0.4	(0.2-0.7)
Low (first tertile)			0.6	(0.5-1.6)
Average women’s education in community (cluster)				
No education			1	
Primary (1-5 years)			1.1	(0.5-2.4)
Secondary (6-10 years)			3.2	(1.6-6.1)
Higher (> 10 years)			11.7	(4.9-28.0)
Average husband’s education in community (cluster)				
No education			1	
Primary (1-5 years)			1.0	(0.4-2.4)
Secondary (6-10 years)			1.5	(0.8-2.9)
Higher (> 10 years)			0.3	(0.2-0.7)
Random effect variances				
	**Model 0** **Null Model**	**Model 1** **Individual**	**Model 2** **Local**	
Variance at local level	1.6^a^	0.8^a^	0.6^a^	
VPC [%]	32.4	20.0	14.7	
Likelihood ratio test	Ref.	2935	80.5	

Abbreviations: VPC, variance partition coefficient; ANC, antenatal care; BC, Baluchistan; KPK, Khyber Pakhtunkhwa; GB, Gilgit-Baltistan. Note: Variation partition coefficient for the null model, individual level and environmental level model are shown also shown. ^a^ *P* < .001

There were inequities between provinces. Women living in Sindh, KPK, and GB were three times more likely to go for ANC. However, urban rural division was not an important determinant at multilevel analysis. Women living in developed communities (indicated by high value of community development index) were more likely to receive ANC. Average women education of the cluster or community had very strong effect on ANC utilization of the individual women, particularly women living in highly educated community with higher level of average education were strong predictor (OR; 12.0) for recommended ANC use. In contrast, in communities with high average level of education of husband lead to no effect or less ANC utilization.

## Discussion

Although Pakistan has made progress in maternal healthcare since 1990, but still maternal health and maternal healthcare utilization is not satisfactory.^1^ To improve maternal health services it is important to know about factors affecting utilization. Pakistan is geographically and culturally diverse country and requires context specific policies and strategies to improve maternal healthcare. First, no national level reports with robust analysis were available to guide about factors of ANC utilization in Pakistan. Secondly, after the 18th constitutional amendment, provinces need context specific environmentally relevant evidence to improve ANC services. This was the first study in Pakistan analyzing ANC (≥4 visits) determinants at national level, exploring factors separately at urban/rural and across different regions and provinces. In addition, interaction in utilization of recommended ANC services between education of women and husband and provinces and urban and rural localities were analyzed. Furthermore, multilevel analysis assessed factors not only at individual and household level but also environmental factors (at cluster level) which influence the individual for seeking ANC.

The study has four-fold impact in identifying the factors for ANC utilization in a developing country: (1) the study identified factors which differ at national level and urban and rural areas. For example, influence of women’s education and health literacy were significantly more in urban than rural areas; ANC utilization was more inequitable among designated urban than rural areas of different provinces as can been seen from interaction effects. Those women who have secondary or higher level of education and living in urban areas of big provinces were synergistically more likely to have recommended ANC utilization (see Supplementary file 1, Table S3); (2) the study identified contextual factors important for provinces. Women’s education significantly increases recommended ANC utilization about 2-7 folds in three provinces only (Punjab, Sindh, GB), while it did not influence it in KPK and BC. Furthermore, husband’s education increased recommended ANC utilization 2-fold in KPK and health literacy increased ANC 3-fold in all areas except KPK province where it had no influence. Also, differential about awareness of family planning (those aware had increase ANC use) occurs in KPK and BC only with much larger difference in BC. Interestingly, residing with husband decreased ANC utilization 1.4 fold in KPK only while it had no influence in other provinces. Distance to health facility decrease ANC in BC only. Increased wealth increased and higher parity decreased ANC utilization, however its influence does not vary across provinces; (3) also identified factors at cluster level which relates to overall context (culture, policies and governance, etc) influencing individual behavior. Living in different provinces influences individual behavior of ANC utilization 1.5-3 fold; developed community itself positively influence individual ANC utilization; higher average women education in a community had strong influence, particularly more than 10 years of average education in a community strongly influences ANC of the individual women (OR; 12.0); interestingly, higher average husband’s education (more than 10 years) decreased ANC utilization about 3 fold. (4) the paper counteract and put into perspective some of the findings of PDHS, which were reported due to inadequate analysis such as location of residence in designated urban or rural areas, is not a strong predictor.

Like many developing countries higher women’s education was uniform predisposing factor for ANC across all regions and provinces.^11,12,24^ Higher women education increased ANC utilization at national level, both in urban and rural areas, and in Punjab, Sindh and GB provinces. Higher education had more pronounced effect on ANC in urban areas and Sindh province. An important finding for this analysis was to note that higher women education had no effect on ANC in KPK and BC. Furthermore, KPK was the only province where higher husband’s education increased ANC. Besides being important predictor at individual level, higher concentration of educated women (average women education of community) had very strong effect on ANC utilization of the individual women, particularly women living in community with higher level of average education (>10 years of formal education) were about 12 times more likely to use ANC. This effect was much higher than individual women education, as individual women higher education increase ANC utilization four times. Overall, we consider that husband’s education has no influence on increasing ANC utilization in Pakistan. Unlike Colombia and Cambodia husband’s education was also not important to influence ANC in MVA for the national level data.^11,25^ However, in communities with high average level of education of husband (>10 years of formal education) led to less ANC utilization. This was a unique and strange finding and need further investigation. But husband’s average education in the community of lower than 10 years has no significant influence on ANC utilization. Our results also showed interestingly strong negative interaction between those women who had higher level of education and their husband’s had primary level of education compared to those couple who had no education.

Overall, health literacy increase ANC utilization across urban rural regions and provinces except KPK province alone. This exception need to be noted and explored further for planning overall maternal services in KPK. Besides, awareness about family planning and exposure to media is a significant predictor only in rural areas. This might be due to uniform access to media and availability and familiarity with reproductive health services in urban areas. So, spreading reproductive health services specific knowledge and dissemination through media in rural areas is still a viable strategy. Awareness about family planning also increase ANC utilization 2-4 fold in KPK and BC provinces only. This finding also suggests that access to health services (ANC and FP) were more inequitable in KPK and BC compared to other provinces.

Small household size is a predisposing factor and increase ANC both at national and subnational level. The larger family size decreased ANC utilization. Although family structure was important predictor in both urban and rural areas, but it was household size that was more important in rural areas and small number of under-5 children in family in urban. Extended families are common in rural and nuclear families are common in urban areas. However, further stratified analysis across provinces reveals that overall household size was important determinant and phenomenon in Sindh province only. While nuclear family system (number of under-5 children) prevails in Punjab and BC provinces and it was not a significant determinant in KPK. This suggests that types of family units have variable distribution across different provinces and it would have implications for health outcome, accordingly.

Previous studies and also PDHS report^2,7^ have found large urban rural differentials for ANC, but in our weighted adjusted analysis this difference was not strong (OR; 1.4). Also, at multilevel analysis living in urban areas did not increase individual ANC utilization. We believe that the reported difference in PDHS was due to crude analysis. The differences seen were more related to differences across provinces and even among the urban and rural areas of provinces. PDHS has used almost two decades old 1998 census circles for choosing urban and rural clusters. Therefore, in order to determine vulnerable population this classification of urban and rural needs to be revised. Furthermore, due to ever growing private sector and contracting out of health services, rural areas may have improved access to maternal care.^26^ Residing in Sindh, Punjab or KPK enabled females for ANC utilization, while urban and rural residence had no significant effect on utilization at environmental level.

Wealth was uniform predictor with similar impact across all provinces, increased ANC utilization with increasing wealth. Wealth index is a robust composite indicator developed and used by PDHS surveys. These findings were consistent with studies from developing countries.^11,27^ Importantly, individual/household wealth strongly influences ANC utilization while average wealth of community (cluster level) had no effect on ANC utilization.

Factors regarding perception of women were weak determinants. Getting money to seek care was an important barrier to ANC in urban areas only, as it may be related to more private sector services.^28^ However, perceived risk due to low parity or to previous pregnancy loss increased ANC utilization across provinces uniformly. Similar findings were reported in other studies and assume that women with higher parity develop confidence and may not be motivated for seeking maternal health services.^11,29^

Inequities in ANC utilization were reported by PDHS report and we also found that ANC of 4 or more visit vary across provinces in the same manner.^30,31^ BC and KPK behave almost similarly while other regions had almost same determinants of ANC. BC had lowest ANC utilization; this may be due to service unavailability. BC is economically underdeveloped and population is widely dispersed therefore providing health services is challenging.^32-34^ Perceived barriers to access due to distance to facility had negative effect on ANC utilization in BC province only. Women’s education was strong predictor in Sindh, Punjab and GB but it was not associated with ANC in BC and KPK. This might be due to cultural differences and more patriarchal society and BC and KPK provinces.^35-37^ Furthermore, higher husband’s education predicted ANC in KPK only, which further supports our assumption of patriarchal society in KPK. Also higher health literacy increased ANC in all provinces except KPK. MWRA who heard of family planning on media were more likely to receive recommended care in BC and KPK. This might be due to uniform availability of information across other provinces and it is not shown as an important predictor but in these provinces (KPK and BC) some sections of the population are exposed to family planning and other were not exposed to family planning methods and those who were exposed were more likely to use ANC. MWRA living with husband were less likely to receive ANC in KPK. In KPK more males have migrated to cities and females who are living alone are doing all work by themselves and they are more autonomous to go for ANC.^38^

## Strengths and Weaknesses

We have done individual and cluster level analysis. However, the analysis can be further strengthened by adding third level (household) but this was not possible in available PDHS data. At several places individual and household variables have been dealt together, for example household wealth. Our multilevel analysis helped to identify community level factors affecting ANC.

Since this was a cross sectional survey, so identified determinants cannot be considered causal. Since healthcare utilization is affected by service availability, but PDHS has no variable related to service availability, therefore, some of the differences seen were probably due to inequitable availability of services, for which we have made some elaboration at several places.

Furthermore, multi-level analysis was not weighted, but minor difference was observed in the ORs with weighted analysis. We found that PDHS has not done major over or under sampling, for example oversampling in few urban areas of BC, ICT, and GB only.^7^

Post-hoc power calculation showed that sample was powered for important determinants at national and sub-national level using weighted and multilevel analysis.

## Policy and Research Implications

The results of the study suggest that there was dire need to develop policies and programs at sub-national level in Pakistan. Gross inequities of ANC utilization exist between and within provinces. This analysis also provides support and push for further implementation of 18th Amendment and strengthening of provincial health systems. The ultimate aim is to have district health system where responsibility and authority lies with the district to have tailor-made service organization. This analysis also raised several research questions which can be further investigated through operational and implementation research. For example, the role of men in providing maternal health services in specific context to KPK. Why women living without husband were more likely to attend ANC services? What should be the new demarcation for vulnerable population and organizing services between urban and rural areas? The analysis also identified strong environmental factors for ANC utilization such as community women education. So, forming community committees of educated women might be used as a vehicle to provide services to the deprived communities. Also, the analysis suggests that there are still specific pockets in KPK and BC, but not all provinces, where mass media campaign is required and would be effective in improving maternal healthcare services.

Some of the inequities identified in the maternal services can be eliminated within short term. Raising heath literacy and mass media campaigns for awareness and developing strategies for women committees for maternal services at district level are short term strategies. However, other identified determinants require long term planning which are related to decreasing the differential between wealth quintile and improving women’s education across provinces.

## Conclusion

Recommended ANC coverage is low in Pakistan and gross inequities were observed among place of residence and socioeconomic status groups. As different set of determinants influence ANC utilization across different regions in Pakistan, so area specific strategies to increase uptake of maternal service utilization is required. This study showed that ANC is influenced by individual and environmental factors. Therefore, environmental factors should also be considered while developing maternal healthcare programs. Since ANC is determined by biological and social determinants so we need to take on board all relevant social sectors like education, social services, transportation along with health for developing comprehensive context specific program.

## Acknowledgements

We gratefully acknowledge the contribution of Dr. Aysha Zahidie, Dr. Adeel Ahmed Khan, and Dr. Unaib Rabbani for their contribution during the early phase of this project.

## Ethical issues

This study is based on secondary analysis of publicly available data hence no ethical approval was required from our institutions. However, the survey followed stringent guidelines for ethical standards detailed in the report.^7^

## Competing interests

Authors declare that they have no competing interests.

## Authors’ contributions

AS has developed the initial concept, analysed the data and wrote the first draft of the manuscript. ZF has reviewed the concept, helped in analysis and reviewed and revised the manuscript. Both authors reviewed and finalized the manuscript.

## Supplementary files

Supplementary file 1 contains Tables S1-S3.

## Authors’ affiliations

^1^Department of Community Medicine, Isra University, Hyderabad, Pakistan. ^2^Department of Community Health Sciences, Aga Khan University, Karachi, Pakistan.

## Key messages

**Implications for policy makers**

- The results of the study suggest that there was dire need to develop health policies and programs at sub-national level in Pakistan. This analysis provides support and push for further implementation of 18th Amendment and strengthening of provincial and district health systems in Pakistan.
- Gross inequities of antenatal care (ANC) utilization exist between and within provinces. ANC utilization is concentrated among educated women (comprise small proportion of population) and in urban areas of large populated provinces (Punjab, Sindh, and Khyber Pakhtunkhwa [KPK]). Provinces with small population (Baluchistan [BC]) and other areas of large provinces are neglected and have low ANC utilization.
- Secondary and higher level of education among women strongly predicts higher ANC utilization, less so for husband’s education. Community level average education has very strong influence to predict higher ANC utilization. The policy makers should also consider the ‘role of men’ in providing maternal health services in specific context to KPK province, as it predicts higher ANC utilization only in this province.
- Urban and rural demarcation is a common tool for policy makers and researchers alike to identify vulnerable population in Pakistan. However, our analysis suggests that urbanization alone is not a strong predictor of ANC utilization. It may imply that new demarcation needs to be made to identifying vulnerable population and organizing services using revised census data.

**Implications for the public**

Antenatal care (ANC) services are a key maternal health indicator. The results of this study have identified gross differences in ANC services between and also within provinces. Secondary or higher level of women education is a key predictor of higher ANC utilization, whether individual or community level. More ANC utilization was seen in urban localities of large provinces (Punjab, Sindh, and Khyber Pakhtunkhwa [KPK]). Context specific strategies can be adopted for improving maternal healthcare and ANC utilization in Pakistan. In specific context to KPK and Baluchistan (BC) provinces, ANC can be improved by mass media campaign and involvement of men could also play a vital role in improving ANC services. Raising heath literacy and mass media campaigns for awareness and developing strategies for women committees for maternal services at district level are also short-term strategies. However, other identified determinants require long term planning which are related to decreasing the differential between wealth quintile and improving women’s education across provinces.
