# Supplementary material for: Inequities in Antenatal Care, and Individual and Environmental Determinants of Utilization at National and Sub-national Level in Pakistan: A Multilevel Analysis
Source: Int J Health Policy Manag. 2018 Jan 30;7(8):699–710. doi: 10.15171/ijhpm.2017.148 (PMC6077283; doi:10.15171/ijhpm.2017.148)
Supplement: Supplementary file 1 — contains Tables S1-S3. [file ijhpm-7-699-s001.pdf]

Table S1. Characteristics of Married Women of Reproductive Age (15-49 years) Who Are Utilizing Recommended And Less Than Recommended Visit for Antenatal Care in Pakistan (n = 7142)

	**Less than recommended (≤3) ANC visits**		**Recommended (≥4) ANC visits**	
	**Weighted %**	**(95% CI)**	**Weighted %**	**(95% CI)**
**1. Predisposing characteristics**				
**Age categories (in years)**				
15-19 years	3.0	(2.3-3.7)	3.0	(2.1-3.9)
20-24 years	19.7	(18.0-21.4)	20.0	(17.8-22.0)
25-29 years	27.2	(25.4-29.0)	30.6	(28.0-33.2)
30-34 years	24.5	(22.8-26.2)	27.4	(25.2-29.6)
35-39 years	15.8	(14.3-17.2)	14.6	(12.8-16.4)
40-44 years	7.4	(6.3-8.5)	3.6	(2.7-4.5)
45-49 years	2.4	(1.8-3.0)	0.9	(0.5-1.3)
**Women's education**				
No education	71.3	(68.3-74.3)	31.3	(27.5-35.0)
Primary (1-5 years)	15.3	(13.3-17.3)	17.9	(15.5-20.3)
Secondary (6-10 years)	11.0	(9.3-12.7)	30.4	(27.4-33.4)
Higher (> 10 years)	2.4	(1.7-3.1)	20.4	(17.3-23.6)
**Husband's education**				

No education	41.5	(38.6-44.3)	19.1	(16.4-21.8)
Primary (1-5 years)	18.2	(16.5-20.0)	13.0	(10.7-15.1)
Secondary (6-10 years)	30.4	(28.0-33.0)	41.3	(38.3-44.3)
Higher (> 10 years)	9.8	(8.3-11.4)	26.7	(23.6-29.7)
**Household size**				
More than 10	26.9	(24.3-29.6)	22.2	(19.5-24.9)
7-10 members	41.2	(39.2-43.4)	37.0	(34.5-39.5)
6 or less	31.8	(29.2-34.3)	40.8	(38.0-43.6)
**Number of U-5 children (Mean, SE)**	2.2	(0.04)	2.0	(0.04)
**Health literacy**				
Low	66.2	(63.1-69.3)	31.7	(28.0-35.3)
High	33.8	(30.7-36.9)	68.3	(64.6-72.0)
**Exposure to media (Radio/TV/Newpaper) (Mean, SE)**	1.2	(0.04)	2.1	(0.04)
**Heard of family planning on media**				
No	81.5	(78.7-84.2)	63.4	(60.0-66.8)
Yes	18.5	(15.8-21.3)	36.6	(33.2-40.0)
**2. Enabling characteristics**				

**Wealth index**				
Poorest	32.0	(26.6-37.4)	8.6	(6.0-11.3)
Poorer	25.4	(22.6-28.0)	11.6	(9.4-13.9)
Middle	21.3	(18.4-24.1)	16.7	(14.3-19.0)
Richer	15.6	(14.0-18.1)	27.3	(24.0-30.5)
Richest	5.8	(4.6-7.0)	35.8	(31.6-40.0)
**Own transport**				
No	70.2	(67.6-72.8)	48.1	(45.0-51.2)
Yes	29.8	(27.2-32.4)	51.9	(48.8-55.0)
**Husband’s occupation**				
Not employed/unskilled	60.0	(56.8-63.3)	36.4	(32.9-40.1)
Skilled/ non-manual	34.7	(31.3-38.2)	51.7	(48.5-54.9)
Professional/technical	5.2	(4.2-6.2)	11.9	(10.0-13.8)
**Women’s employment status**				
Not employed	67.4	(64.6-70.2)	66.3	(62.5-70.0)
Employed	32.6	(29.8-35.4)	33.7	(30.0-37.5)
**Residing with husband**				
No	13.2	(11.2-15.2)	13.1	(10.6-15.6)
Yes	86.8	(84.8-88.8)	86.9	(84.4-89.4)

**Women's autonomy**				
Low	50.6	(48.3-52.9)	49.2	(46.4-51.9)
High	49.4	(47.1-51.7)	50.8	(48.1-53.5)
**Getting permission**				
Problem	77.6	(74.5-80.6)	77.0	(74.1-79.9)
Not a problem	22.4	(19.4-25.5)	23.0	(20.1-25.9)
**Getting money for treatment**				
Problem	63.0	(59.6-66.5)	62.7	(58.8-66.6)
Not a problem	36.9	(33.4-37.2)	37.3	(33.4-41.2)
**Distance to health facility**				
Problem	55.3	(51.8-58.9)	54.8	(50.7-59.0)
Not a problem	44.6	(41.1-48.2)	45.2	(41.0-49.3)
**Having to take transport**				
Problem	50.1	(46.3-53.9)	51.9	(48.0-55.8)
Not a problem	49.9	(46.1-53.7)	48.1	(44.2-52.0)
**Going alone for treatment**				
Problem	38.2	(35.2-41.1)	40.8	(37.5-44.0)
Not a problem	61.8	(58.9-64.7)	59.2	(55.9-62.5)
3. **Need based characteristics**				

**Parity**				
5 or more children	38.5	(36.4-40.5)	21.6	(19.3-23.9)
3-4	30.2	(28.2-32.1)	30.2	(27.9-32.5)
2 or less	31.4	(29.6-33.1)	48.2	(45.5-50.8)
**Wanted child**				
No	19.2	(17.3-21.1)	18.8	(16.5-21.2)
Yes	80.8	(78.9-82.7)	81.1	(78.8-83.5)
**Pregnancy loss**				
No	67.0	(65.1-68.9)	63.7	(61.2-66.2)
Yes	33.0	(31.0-34.8)	36.3	(33.8-38.8)
**4. Health seeking behaviour**				
**Use of family planning method**				
No/traditional method	73.0	(70.8-75.2)	73.0	(70.7-75.3)
Modern	27.0	(24.8-29.2)	27.0	(24.7-29.3)
**5. Environmental factors**				
**Region**				
Baluchistan	6.7	(4.0-9.3)	1.6	(0.9-2.3)
KPK	18.6	(13.6-23.5)	10.4	(7.6-13.2)
Gilgit Baltistan	0.8	(0.5-1.2)	0.6	(0.3-1.0)
Punjab	53.2	(46.1-60.2)	57.3	(50.8-63.8)

Sindh	20.6	(15.3-25.9)	29.0	(23.2-34.7)
ICT	0.1	(0.06-0.2)	1.0	(0.6-1.3)
**Place of residence**				
Rural	82.5	(79.2-85.9)	49.3	(44.0-54.6)
Urban	17.4	(14.1-20.8)	50.7	(45.4-56.0)
**Community development index**				
Low	81.8	(78.4-85.2)	45.1	(39.8-50.3)
High	18.2	(14.8-21.6)	55.0	(49.7-60.2)

Abbreviations: ANC, antenatal care; KPK, Khyber Pakhtunkhwa; SE, standard error; ICT, information and communications technology.

Table S2. Adjusted (Multivariate Analysis) Factors Affecting Recommended ANC Utilization Stratified by Rural and Urban Residence, Among Women in Pakistan – PDHS 2012-13.

	**Rural (n=4032)**		**Urban (n=3110)**	
**Characteristics**	**OR**	**95%CI**	**OR**	**95%CI**
**Predisposing characteristics**				
**Women's education**				
No education	1		1	
Primary (1-5 years)	1.4	(1.0-2.0)	1.4	(1.0-1.9)
Secondary (6-10 years)	1.6	(1.2-2.3)	2.6	(1.9-3.7)
Higher (> 10 years)	2.6	(1.5-4.6)	6.1	(3.5-10.6)
**Household size**				
More than 10	1			
7-10 members	1.1	(0.9-1.4)	-	
6 or less	1.5	(1.1-1.9)		
**Number of U-5 children (per child increase)**	-	-	0.8	(0.7-0.9)
**Health literacy**				
Low	1		1	
High	1.4	(1.1-1.8)	1.6	(1.2-2.1)

**Heard of family planning on media**				-
No	1		-	
Yes	1.5	(1.2-1.9)		
**Enabling characteristics**				
**Region**				
Baluchistan	1		1	
KPK	1.3	(0.8-2.1)	2.3	(1.4-3.6)
Gilgit Baltistan	3.9	(1.9-8.1)	6.3	(3.4-11.7)
Punjab	1.7	(1.1-2.6)	3.6	(2.4-5.4)
Sindh	2.9	(1.8-4.7)	6.0	(3.9-9.3)
ICT	5.5	(3.2-9.4)	8.6	(4.9-15.1)
**Wealth index**				
Poorest	1		1	
Poorer	1.7	(1.3-2.3)	1.1	(0.4-2.9)
Middle	2.2	(1.5-3.1)	3.0	(1.3-7.0)
Richer	3.4	(2.3-5.1)	4.2	(1.7-10.3)
Richest	7.1	(4.4-11.4)	7.4	(3.1-17.7)
**Need based characteristics**				
**Parity**				
5 or more children	1		1	
3-4	1.0	(0.7-1.3)	1.2	(0.8-1.8)
2 or less	1.4	(1.1-1.9)	1.9	(1.2-3.0)

**Pregnancy loss**						
No			1		1	
Yes			1.6	(1.3-1.9)	1.4	(1.0-1.8)
**Getting money for health care**	-	-	-			
Problem				-	1	
Not a problem					1.6	(1.2-2.0)

Abbreviations: ANC, antenatal care; KPK, Khyber Pakhtunkhwa; OR, odds ratio; ICT, information and communications technology.

Table S3. Interaction Between Urban Areas, Provinces and Educational Level of Women and Husband in Multivariate Adjusted Model for Recommended ANC Utilization in Pakistan (PDHS-2012-13).

**Interaction between provinces and urban areas**	**OR (95% CI)**	
Baluchistan*Rural	1.0	
Punjab*Urban	2.4	(1.3- 4.4)
Sindh*Urban	2.3	(1.2- 4.5)
KPK*Urban	2.0	(1.0- 3.7)

**Interaction between women education and provinces**		
Baluchistan* Woman's None(no education)	1.0	
Punjab* Woman's Secondary education	2.2	(1.0-4.8)
Punjab* Woman's Higher education	7.1	(2.1-23.7)
Sindh* Woman's Secondary education	2.2	(1.0-5.0)
Sindh* Woman's Higher education	11.6	(3.4- 40.1)
KPK* Woman's Higher education	4.6	(1.4-15.4)
GB* Woman's Secondary education	5.1	(1.9-13.8)
ICT* Woman's Secondary education	2.9	(1.1-7.4)
ICT* Woman's Higher education	11.5	(2.7-49.9)
**Interaction between husband's education and provinces**		
Baluchistan*Husband's No education	1.0	
Punjab* Husband's Higher education	2.3	(1.1-4.8)
Sindh* Husband's Higher education	3.2	(1.5-6.8)
KPK* Husband's Higher education	3.6	(1.6-8.0)
GB* Husband's Higher education	3.8	(1.2-12.1)

ICT* Husband's Higher education	5.4	(1.6-18.3)
**Interaction between woman's education and urban areas**		
Rural* Woman's no education	1.0	
Urban* Woman's Secondary education	1.7	(1.1-2.6)
Urban * Woman's Higher education	2.6	(1.2-5.5)
**Interaction between woman's education and husband's education**		
Women's No education*Husband's no education	1.0	
Woman's higher education*Husband's primary education	0.09	(0.02-0.6)

Abbreviations: ANC, antenatal care; KPK, Khyber Pakhtunkhwa; OR, odds ratio; ICT, information and communications technology; GB, Gilgit-Baltistan.
